# Nonrigid Registration of Brain Tumor Resection MR Images Based on Joint Saliency Map and Keypoint Clustering

**DOI:** 10.3390/s91210270

**Published:** 2009-12-17

**Authors:** Zhijun Gu, Binjie Qin

**Affiliations:** Department of Biomedical Engineering, School of Life Sciences & Biotechnology, Shanghai Jiao Tong University, Shanghai 200240, China; E-Mail: gzj0126@gmail.com

**Keywords:** nonrigid registration, tumor resection, keypoint, outlier, local large deformation, brain shift, clustering

## Abstract

This paper proposes a novel global-to-local nonrigid brain MR image registration to compensate for the brain shift and the unmatchable outliers caused by the tumor resection. The mutual information between the corresponding salient structures, which are enhanced by the joint saliency map (JSM), is maximized to achieve a global rigid registration of the two images. Being detected and clustered at the paired contiguous matching areas in the globally registered images, the paired pools of DoG keypoints in combination with the JSM provide a useful cluster-to-cluster correspondence to guide the local control-point correspondence detection and the outlier keypoint rejection. Lastly, a quasi-inverse consistent deformation is smoothly approximated to locally register brain images through the mapping the clustered control points by compact support radial basis functions. The 2D implementation of the method can model the brain shift in brain tumor resection MR images, though the theory holds for the 3D case.

## 1. Introduction

Image registration is an important enabling technology for neuronavigation [1, 2] due to its mapping pre-operative images to the patient's anatomy in physical space and augmenting the intra-operative images with the pre-operative image. Image registration can be classified into intensity- and landmark/feature-based methods [3]. It can be considered as an optimization problem, posed as finding the optimal transformation *T* between the reference image *I_R_* and the floating image *I_F_* to maximize a defined similarity measure such as mutual information (MI) [4, 5]. The transformation space includes rigid and nonrigid that compensates for deformations. During the last decade, nonrigid registration of MR brain images has attracted much attention at the brain shift estimation [1, 2] in image-guided neurosurgery.

The key challenge for the nonrigid registration of pre- and intra-operative MR images is to compensate for the local large tissue distortion caused by the tumor resection. The local large tissue deformations with irregular shapes violate the usual assumption of smoothness of the deformation fields. An additional challenge exists when the unmatchable outlier features (*i.e.*, a tumor in the preoperative image may not even exist in the intra-operative image) are introduced. Moreover, the local large deformation, sharp geometric difference between the pre- and intra-operative MR images and the confounding effects of edema and tumor infiltration, render the outlier problem more intractable.

To reject the outliers, many intensity-based registration approaches are proposed including M-estimator [6] or mixture-based similarity measure [7], graph-based multifeature MI [8], least-trimmed square based outlier rejection [1], consistency test [9, 10] and intensity modification [11]. However, the intensity similarity does not necessarily mean anatomical similarity and easily suffers from local and biased maxima [12–15] when outliers are presented in images. Additionally, by forcibly matching non-corresponding structure features, the extra flexibility of the complex deformation in intensity-based methods may make the results unpredictable and less reliable. To somewhat alleviate these problematic aspects, modifications have been added in intensity-based methods to include higher level feature information such as landmark [16, 17]. Despite these modifications, the presence of local large deformation and the outliers still remains an unresolved problem for most intensity-based methods.

Recently, landmark-based registration methods using local invariant features, such as salient region features [18–20], multi-scale Laplacian blob [21, 22], SIFT keypoint [23–28], attribute vector [29–31] and multiscale wavelet-based region [32], have been proven effective than intensity-based methods in compensating for the local deformations around small anatomical structures. The SIFT detector has been successfully applied to various applications such as face recognition [33] and medical image registration [24–27]. In image registration, the SIFT keypoints [24, 27] localized at specific anatomical structures could be automatically selected for the adaptive setup of the irregular control point grids for the local deformation of specific anatomical structures. Without tedious manual selection of control points, the adaptive setup of irregular control point grids could alleviate the computational cost and the registration inaccuracy that are related to the regular grids [34] of control points arranged for the local large deformation at the tumor resection region. In general, it is often difficult to correctly match local keypoints [26, 28] by using only the similarity between SIFT descriptors when complex local deformation and outliers exist in brain images. The deformation invariant local feature descriptor was presented in [29], however this topic is beyond the scope of this paper.

Although the results of these above methods clearly demonstrate the power of local invariant feature-based nonrigid deformations, the desired landmark-based registration algorithm should establish robust control point correspondence to accurately model the complex local deformation around the tumor resection region. To find robust point correspondence, some approaches are proposed including soft correspondence detections [20, 35], joint clustering-matching strategy [36] and modeling point sets by kernel density function [37]. Compared with the classical template matching, the iterative closest point [38] and the correspondence by sensitivity to movement [39], the self-organizing map [40] algorithm was considered in [41] to be the most effective method in 2D feature point correspondence detection. However, these methods do not consider the complexity of correspondence detection in the context of local large structure distortion combined with the outliers. In this work, we first compute the global correspondence between the contiguous matching areas of normal tissues and the tumor regions in the two images by using MI-based rigid registration method. The global correspondence is then used to introduce the cluster correspondence between the paired pools of DoG keypoints [23] that are detected and clustered at those contiguous matching areas in the two images. *An important contribution is that we have proposed the cluster-to-cluster correspondence can be introduced as a useful constraint for the local point correspondence detection within the paired pools of keypoints*.

To make the rigid registration more accurate and robust for matching brain tumor resection images, we use joint saliency map (JSM), first proposed in our previous work [15], to highlight the corresponding salient structures in the two images, and emphatically group those salient structures into the smoothed compact clusters in the weighted joint histogram for MI based registration. *Another important contribution is that we have demonstrated the JSM can be combined with the keypoint clustering for nonrigid landmark-based registration scheme, ensuring that the structural mismatches at the tumor regions could be detected and the underlying outlier DoG keypoint could be rejected automatically*. Specifically, after implementing keypoint clustering, we identify the pair of tumor resection clusters in both images owing to its average JSM value being below a threshold value. We then remove the outlier keypoints inside the paired tumor areas, use the cluster-to-cluster correspondence to guide the point-to-point correspondence detection of the significant keypoints within the paired matching keypoint groups. Once the significant keypoint sets are selected and classified into a number of local adaptive setup of irregular control point grids, the nonrigid local registration is carried out within deterministic annealing framework with the implementation of quasi-inverse consistency [16, 42] radial basis functions (RBF) [16, 40, 43] as a warping method.

The proposed algorithm has been applied to the nonrigid registration of 2D brain tumor resection MR images. Experimental results show that, compared to other classic nonrigid registration methods, our proposed method can provide better robustness and higher accuracy for the registration of brain tumor resection images. The rest of this paper is organized as follows. Section 2 briefly summarizes the definition of JSM and its application in global rigid registration, which are the same as in [15]. Section 3 integrates the JSM into the keypoint clustering for robust correspondence detection with outlier rejection, and implements the irregular control point setup for RBF-based local nonrigid registration within a deterministic annealing framework. In Section 4, using clinical brain tumor resection images, we report preliminary experiment results to identify the registration performance on accuracy and robustness. The conclusion and future work are discussed in Section 5.

## 2. Global Registration Based on Joint Saliency Map

Since 1995 [4, 5], mutual information (MI) has proved effective in intensity-based image registration. For a reference image *I_R_* and a floating image *I_F_* with intensity bins *r* and *f*, the MI between *I_R_* and *I_F_* is defined as:

(1) $$\text{MI}=H(I_{R})+H(I_{F})-H(I_{R},I_{F})$$

where *H*(*I*) = −Σ*_i_p*(*i*) log *p*(*i*) and *H* (*I_R_, I_F_*) = −Σ*_r,f_p* (*r, f*) log *p* (*r, f*) are the entropy of the intensities of image *I* and the joint entropy of the two images, *p*(*i*) is the intensity probabilities with *p* (*r*) = Σ*_f_p* (*r, f*) and *p* (*f*) = Σ*_r_p* (*r, f*), *p* (*r, f*) is the joint intensity probabilities estimated by a joint histogram *h* (*r, f*).

**Figure 1.**
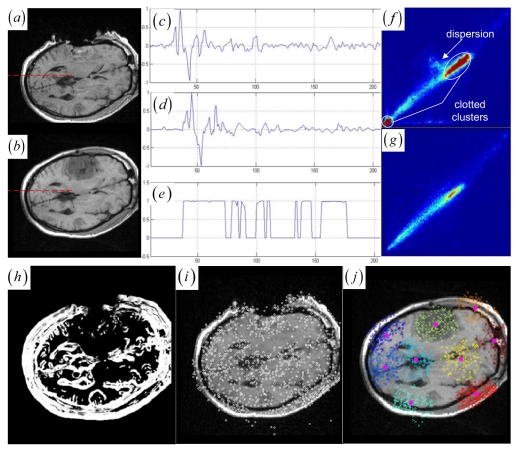
(a)–(b) Intra- and pre-operative MR images with tumor resection. (c)–(d) Gradient value profiles of the lines in (a)–(b), which are marked as dashed lines. (e) JSM value profiles of the lines in (a)–(b). (f) Joint histogram dispersion with two clotted clusters (dark red in pseudo color). (g) The JSM-weighted joint histogram with smoothed compact clusters for (a)–(b). (h) JSM for the two images in (a)–(b) with low JSM values at the tumor resection area. (i) The intra-operative MR image and the circle marked DoG keypoints. (j) The pre-operative image and resultant keypoint clustering with circle marked floating keypoints and cross marked reference keypoints. Different colors mean different clusters.

MI-based registration methods take advantage of the fact that properly registered images usually correspond to compactly-clustered joint histograms [44]. They measure the joint histogram dispersion by computing the entropy of the joint intensity probabilities. When the images become misregistered, the compact clusters become disperse sets of points in the joint histogram. The JSM not only emphatically groups the corresponding salient pixel pairs into the compact histogram clusters for robust global rigid registration, but also determines the structural mismatches for subsequent local nonrigid registration. The idea of JSM is demonstrated schematically in Figure 1h, where the high fractional joint saliency values (between 0 and 1) are assigned to the corresponding salient pixel pairs rather than the structural mismatches (tumor areas) and the homogeneous pixel pairs.

### 2.1. Joint Saliency Map

Noting that many techniques have defined the saliency of image to enhance the image pixels we are interested in, *i.e.*, using edge gradient, corner, salient regions [18] and keypoint [23]. Nevertheless, gradient is a local feature and sensitive to noise, corner and keypoint saliency are not to be defined for each pixel, and salient regions suffers from high complexity. In this study, we first use multi-scale saliency map [45] *S_l_*(**v**) = Σ**_u_**_∈_*_N_***_v_** (*I_l_* (**v**) − *I_l_* (**u**))^2^, where *N***_v_** is the 1-pixel radius circular neighborhood of the pixel position **v** = (*x, y*) at scale *l, S_l_*(**v**) is the local saliency computed from the intensity *I_l_*(**v**) of the pixel position **v** in the Gaussian image pyramid [46] at scale *l, I_l_*(**u**) is the intensity of the pixel in the *I_l_*(**v**)'s neighborhood. The final saliency map *S*(*x, y*) is reconstructed by summing up all the saliency maps at the coarser scales.

In the second step, a principal axis analysis of regional saliency distribution assigns the regional saliency vector to each pixel based on the inertia matrix:

(2) $$\text{M}=\left[\begin{array}{cc} \varphi_{20} & \varphi_{11}\\ \varphi_{11} & \varphi_{02} \end{array}\right]$$

where *φ_mn_* = Σ(*x* − *g_x_*)*^m^*(*y* − *g_y_*)*^n^S*(*x, y*), (*g_x_, g_y_*) = (*ω*_10_*/ω*_00_, *ω*_01_*/ω*_00_), *ω_mn_* = Σ*x^m^y^n^S*(*x, y*) are the central (*m, n*)- moment, the centroid and the (*m, n*)- moment of the regional saliency distribution *S*(*x, y*) in a 5.5-pixel radius circular neighborhood around the center pixel. This regional saliency distribution describes a 2D regional salient structure, within which the two eigenvectors of the *M* represent the orthogonal coordinate system. The first eigenvector referred as the RSV is enough to represent the regional salient structure around a pixel due to its storing the regional information about the orientation of the salient structure.

Given the two RSVs s*_r_* and s*_f_* at an overlapping location **v**, the JSM is constructed to indicate the corresponding salient structures in the two images by computing the inner product of two RSVs. The essential idea of JSM is always valid in practice in image registration: for two precisely aligned images, the majority of the corresponding pixel locations are very likely to produce RSVs with similar orientations. This is because the two images under registration fundamentally depict the same image structures. As a result, the RSVs of the corresponding pixel locations from two images could present relatively coincident orientations in general. Therefore, the angle *γ* between the two vectors is simply calculated, making cos *γ* the scalar measure of the JSM value *w* (**v**) = cos *γ* (s*_r_*, s*_f_*) = 〈s*_r_*, s*_f_* 〉/‖s*_r_*‖ · ‖s*_f_*‖.

A JSM value near one suggests that the underlying pixel pair comes from the corresponding spatial structures. Contrarily, a JSM value near zero indicates that the pixel pair originates from either the outliers or a homogeneous region. To speed up the rigid registration procedure without reducing accuracy, the registration only uses the salient pixel pairs with large saliency values. The pixel with a small saliency value below a fixed threshold value (10 percent of the maximum saliency value) is assigned zero JSM value directly. Generally, the JSM would primarily respond to the high-gradient common edges in both images if a high threshold is chosen to exclude more pixels from estimating the JSM. However, as in the Figure 1c–e representing the image gradient and the JSM profiles of the same line at the two registered images (see Figure 1a,b), the JSM (see Figure 1e) consistently captures the corresponding regional salient structures in the two images while the image gradient features (see Figure 1c,d) are very noisy and do not agree with each other at each overlapping location.

### 2.2. JSM-Weighted Mutual Information

The accurate rigid registration can be iteratively achieved by maximizing the MI between the corresponding salient structures that are highlighted by the JSM. The JSM is updated with the transformation *T* changing the overlapping area of the two images during the rigid registration. Specifically, the JSM weights each overlapping pixel pairs for the joint histogram so that the contribution of the interpolated floating intensity *f* (**v***_f_*) to the histogram is weighted by the *w* (**v**) of the JSM. For example, if using nearest neighbor or linear interpolation, the value *w*(**v**) should be added to the histogram entry *h*(*r, f*) for the overlapping intensity pair (*r, f*). In the JSM-weighted joint histogram, the histogram entries related to the outliers and homogeneous regions have little impact on the histogram dispersion. Furthermore, each histogram entry for the corresponding salient structures is the sum of smoothly varying fractions of one. As a result, the smoothed compact clusters (see Figure 1g) in the neighboring bins for the corresponding salient structures is introduced to solve the outlier-induced dispersion and the undesired clotted clusters caused by the homogeneous regions (see Figure 1f) in the histogram.

## 3. Local Nonrigid Registration Based on Keypoint Clustering

Given the globally registered images and the affiliated JSM (see Figure 1a,b,h), there is a global correspondence between the paired contiguous matching areas from the corresponding anatomical tissues and tumor regions in the two images. Moreover, the deformations of the corresponding anatomical tissues and tumor regions occur locally in the paired matching areas. For example, the tumor-induced local large deformation occurs in the paired corresponding tumor regions (see Figure 1a,b). To facilitate characterization of those local deformations, keypoints belong to those contiguous matching areas can be further grouped into paired keypoint clusters, since they presumably represent the local deformations of the paired contiguous matching areas in the brain tumor resection images.

### 3.1. EM Algorithm-Based Keypoint Clustering

Ideas related to clustering based control point setup was first suggested by Chui *et al.* [36]. The cluster centers of point sets is provided for a concise representation of the original point data and is used as control points for deformation. Recently, clustering-based registration of brain white matter fibers has been developed in [47]. In this work, we automatically detect 2D stable DoG keypoints by adopting an open implementation [48] of the SIFT detector [23]. Figure 1i shows that the many DoG keypoints are extracted from the intra-operative MR images. Subsequently, keypoint clustering is introduced so that the paired keypoint groups can be provided for a point cloud representation of the paired contiguous matching areas in the two images. This could introduce the intermediate cluster-to-cluster keypoint correspondence which represents a useful constraint on the local point-to-point keypoint correspondence detection for the local nonrigid registration.

In order to divide the keypoints into groups, we make use of the EM algorithm [49] to determine the maximum likelihood parameters of a mixture of *K* Guassian components in the keypoint space. The EM algorithm is used for finding maximum likelihood parameter estimates when there is missing or incomplete data. In our case, the missing data is the Gaussian cluster to which the points in the keypoint space belong. Assume that we are using *K* Gaussians in the mixture model. The probability density function of the mixture model can be stated as follows:

(3) $$p(\text{y}|\theta )=\sum_{i=0}^{K}\alpha_{i}p_{i}(\text{y}|\theta_{i})$$

where **y** = (*x, y*) is a coordinate vector of a 2D keypoint, *i* is the cluster index, the *α_i_*'s represent the mixing weights ( $(\sum_{i=0}^{K}\alpha_{i}=1)$, *θ* represents the collection of parameters (*α*_1_,…, *α_K_, θ*_1_,…, *θ_K_*), and *p_i_* is a two-dimensional Gaussian density parameterized by *θ_i_* = (*μ_i_*, Σ*_i_*) (*i.e.*, the means and covariance):

(4) $$p_{i}(\text{y}|\theta_{i})=\frac{1}{2\pi {\left|\sum_{i}\right|}^{1/2}}e^{-\frac{1}{2}{\left(\text{y}-\mu_{i}\right)}^{T}\sum_{i}^{-1}(\text{y}-\mu_{i})}$$

The first step in applying the EM algorithm to the Gaussian mixture problem is to initialize *K* mean vectors *μ*_1_,…, *μ_K_* and *K* covariance matrices Σ_1_,…,Σ*_K_* to represent each of the *K* groups. We initialize these parameters using improved K-means clustering (We will return to the details of choosing *K* and the initial parameters shortly.). On subsequent restarts of the EM iteration, we have found that using improved K-means clustering initialization yields good results with fast convergence. The update equations take on the following form:

(5) $$\alpha_{i}^{\text{new}}=\frac{1}{N}\sum_{j=1}^{N}p\;(i|{\text{y}}_{j},\theta^{\text{old}})$$

(6) $$\mu_{i}^{\text{new}}=\frac{\sum_{j=1}^{N}{\text{y}}_{j}p\;(i|{\text{y}}_{j},\theta^{\text{old}})}{\sum_{j=1}^{N}p\;(i|{\text{y}}_{j},\theta^{\text{old}})}$$

(7) $$\sum_{i}^{\text{new}}=\frac{\sum_{j=1}^{N}p\;(i|{\text{y}}_{j},\theta^{\text{old}})({\text{y}}_{j}-\mu_{i}^{\text{new}}){\left({\text{y}}_{j}-\mu_{i}^{\text{new}}\right)}^{T}}{\sum_{j=1}^{N}p\;(i|{\text{y}}_{j},\theta^{\text{old}})}$$

where *N* is the total number of keypoints, and *p* (*i*| y*_j_, θ^old^*) is the probability that Gaussian *i* fits the keypoint y*_j_*, given the data *θ^old^*:

(8) $$p(i|{\text{y}}_{j},\theta^{\text{old}})=\frac{\alpha_{i}p_{i}({\text{y}}_{j}|\theta_{i}^{\text{old}})}{\sum_{k=1}^{N}\alpha_{k}p_{k}({\text{y}}_{j}|\theta_{i}^{\text{old}})}$$

The above update equations are iteratively computed until the log likelihood $\log\prod_{j=1}^{N}p({\text{y}}_{j}|\theta )$ increases by less than 1 percent from one iteration to the next.

We have thus far not discussed how to choose *K* and the initial parameters (*α*_1_,…, *α_K_, θ*_1_,…*, θ_K_*) of the EM algorithm. Choosing the number of clusters in a Gaussian mixture model [50] is a famous unsolved problem. Ideally, we would like to choose the number of clusters and those initial parameters that best suit the natural characteristic patterns of keypoint groups around the salient anatomical structures. Note that those keypoint groups may include a number of spatially disjoint regions in the brain images, and one readily available pattern representing the distribution for the random variable (*x, y*) of the keypoints is the joint histogram. It is generally believed that the number of peaks marked on the histogram may correspond to the number of Gaussians while the valleys specify the means and variances of Gaussian mixture models. Based on this knowledge, we can automatically detect the peaks and valleys in a smoothed histogram [51] as follows: (1) exponentially smooth the keypoint histogram to remove noisy peaks and valleys. The simplest form of exponential smoothing for each direction (*x, y*) of the histogram is given by the formula:

(9) $$\begin{array}{l} E_{1}=H_{1}\\ E_{j}=\rho H_{j}+(1-\rho )E_{j-1}=E_{j-1}+\rho (H_{j}-E_{j-1}) \end{array}$$

where *ρ* is the smoothing factor, and 0 < *ρ* < 1, *H_j_* is *j*th the histogram value. We choose the desired *ρ* for minimizing the mean square error (*MSE*) according to the criterion:

(10) $$\rho =\arg\underset{\rho }{\min}MSE=\arg\underset{\rho }{\min}\left(\frac{1}{N}\sum_{i,j=1}^{\max i,\max j}{\left(H_{ij}-E_{\left(i-1)(j-1\right)}\right)}^{2}\right)$$

(2) detect peaks and valleys. A point *H_i_* in histogram is a peak or a valley if and only if *H_i_* is the largest or the smallest among its 8 neighbors; (3) remove pseudo peaks (or valleys) if the distance between neighboring peaks (or valleys) is less than a distance threshold. A reasonable distance threshold is (*d*_max_ − *d*_min_)*/*2 < *D_threshold_* < *d*_max_; (4) reject each keypoint which is far from any peaks obtained from step 3 under the distance threshold (1-2 times *D_threshold_*); (5) compute the number of peaks and the initial parameters of Gaussian mixture model. If the set of peaks and valleys are *P* = {*t*_0_, *t*_1_,…, *t_l_*}, *V* = {*v*_0_, *v*_1_,…, *v_l_*}, the initial parameters are given by the formula:

(11) $$\Omega_{j}^{0}=\sum_{i=v_{j}-1}^{v_{j}}h_{i}$$

(12) $$\mu_{j}^{0}\frac{1}{\Omega_{j}^{0}}\sum_{i=v_{j}-1}^{v_{j}}ih_{i}$$

(13) $$\sum_{j}^{0}=\frac{1}{\Omega_{j}^{0}}\sum_{i=v_{j}-1}^{v_{j}}(i-\mu_{j}^{0})h_{i}$$

where *h_i_* is the probability that a pixel has a gray level intensity *i*.

Given a set of keypoints (**y**_1_, **y**_2_,…, **y***_N_*) and the initial peak locations which are used as initial *K* means $\mu_{1}^{1},\ldots ,\mu_{K}^{1}$, K-means clustering is started to partition the *N* observations into *K* sets (*K* < *N*) *C* = {*C*_1_,*C*_2_,…,*C_K_*} so as to minimize the within-cluster sum of squares:

(14) $$\arg\underset{C}{\min}\sum_{i=1}^{K}\sum_{{\text{y}}_{j}\in C_{i}}\;{\left\|{\text{y}}_{j}-\mu_{\text{i}}\right\|}^{2}$$

where *μ_i_* is the mean of *C_i_*. The algorithm proceeds by alternating between two steps. For Step 1, every point is assigned to the cluster with the closest mean. In Step 2, the centroid is computed for each set. The algorithm is stopped when the assignments of the keypoints no longer change. The output of the K-means clustering is the $\left(\mu_{1}^{1},\ldots ,\mu_{K}^{1}\right)$ and the *C* = {*C*_1_,*C*_2_,…,*C_K_*}, on which we obtain the mixing weights by computing $\alpha_{i}^{1}=\text{card}\;(C_{i})/N$. The covariance matrices $\sum_{1}^{1},\ldots ,\sum_{K}^{1}$ could be obtained by computing the within-cluster statistics of keypoints. These values provide the ideal initial parameters of the EM algorithm.

The results of the EM clustering method are probabilistic. This means that every keypoint belongs to all clusters, but each assignment of a keypoint to a cluster has a different probability. Given the resultant parameters (*α*_1_,…, *α_K_, θ*_1_,…, *θ_K_*) for a mixture of Gaussians, we search for the cluster index *z_i_* that meets *z_i_* = arg max *α_k_p_k_* (**y***_i_* | *θ_k_*) to estimate the Gaussian cluster to which the each keypoint **y***_i_* belongs. Figure 1j shows that the circles representing floating keypoints and the crosses depicting the reference keypoints are classified into nine corresponding clusters.

### 3.2. Outlier Rejection and Correspondence Detection

**Figure 2.**
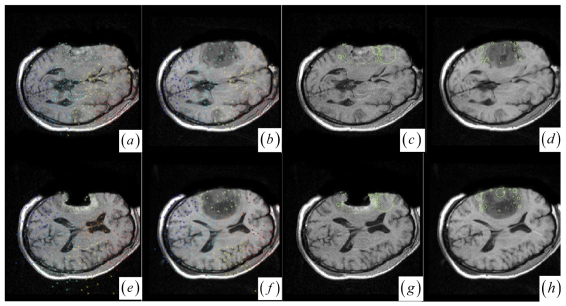
(a)–(b) and (e)–(f) Clustered significant keypoints in the two images. (c)–(d) and (g)–(h) Boundary significant keypoints around tumor resection regions with circles defining the scale measures of the points.

Note that if one cluster pair of tumor regions suffers from the large structural mismatches with local large deformation and outliers, this cluster pair will bring together many keypoints and small average JSM value *ζ_k_* (computed as *ζ_k_* = Σ*w* (**y***_i_*)*/card* (*C_k_*) (**y***_i_* ∈ *C_k_*)). Therefore, the keypoint cluster of tumor region in each image can be localized if its *ζ_k_* is below a threshold value (*ζ_threshold_* = 0.4). In Figure 1j and Figure 2a,b, the keypoint clusters in both images are sorted according to the average JSM values {0.266, 0.404, 0.494, 0.533, 0.560, 0.565, 0.590, 0.639, 0.734}. The tumor resection clusters with *ζ*_1_ = 0.266 could be automatically detected from the nine clusters. Figure 2e,f shows that the two images have ten clusters with the tumor resection cluster having 0.234 average JSM value. Furthermore, the keypoint clusters of tumor regions in both images could be regarded as an ellipse model based on the mean and variation of the cluster, and then the outlier keypoints inside the ellipse are removed while the boundary significant keypoints (Figure 2c,d,g,h) are saved for subsequent correspondence detection, *i.e.*, delete **x** = (*x, y*) if [ (*x* − *μ_x_*)^2^*/*(*κσ_x_*)^2^ + (*y* − *μ_y_*)^2^*/*(*κσ_y_*)^2^] < 1 with *κ* = 0.1. After outlier keypoint rejection, we approach the control point setup and related correspondence detection as follows.

In general, correspondences are found by choosing the two points with the optimum similarity measure (such as mutual information and cross correlation) between the points' surrounding regions, but this template matching method can give unsatisfactory correspondence due to the large number of keypoints detected in each image and the region around each keypoint being too small to include sufficient information. To enhance the confidence of template matching-based correspondence detection, we use two steps to find the robust correspondence:

1. Choose the significant keypoints for irregular control point setup. The SIFT keypoint detector has assigned a location and a scale to each stable DoG keypoint. The scale defines the saliency measure of each keypoint such that the keypoint with a large scale could be identified at the same location in the noisy pre- and intra-operative MR brain images. Based on the above consideration, a keypoint with the largest scale measure within a neighborhood could be saved as the significant keypoint in a cluster. In Figure 2a,b,e,f, the significant keypoints selected from each cluster well represent the irregular control point setup in the salient structures. Figure 2c,d,g,h shows the boundary significant keypoints at the tumor resection clusters.
2. Use the cluster-to-cluster correspondence and inverse consistent correspondence calculation to enhance the confidence of MI-based template matching. In detail, we use a local MI (see the details in the following section) to match a reference point **r***_i_* in cluster *k* to the floating points within a certain search radius *δ_k_* in the corresponding cluster at floating image. The floating points, together with their similarity values to the reference point, define a forward search map of the inverse consistent correspondence detection. The floating point with the maximum local MI in the forward search map can be appointed as a candidate corresponding point **f***^i^*. For inverse consistency, to mach a floating point **f***_i_* in cluster *k*, a backward search map defines a candidate corresponding point **r***^i^*. When the constraint of inverse consistent correspondence detection is satisfied, *i.e.*, **r***_i_* = **r***^i^* and **f***_i_* = **f***^i^*, the point pair (**r***_i_*, **f***_i_*) is accepted as a corresponding control point pair.

In recent years, increased attention has been paid to the local similarity measure of small image regions for local nonrigid registration [52–54]. Among various local similarity measures, the information-theoretic local similarity measures such as local MI [52] have proved effective in nonrigid registration. Noting that a local MI between two corresponding areas can be low if the two local salient areas are mismatched, or if either of the corresponding areas is featureless and therefore has low entropy (This is because MI is bounded by the entropies of the individual areas, *i.e.*, MI (*a, b*) ≤ min {H(*a*), H(*b*)}), we solved this ambiguity by adopting the idea of local mismatch measure [52] to evaluate the local MI (LMI) as LMI = MI (*a, b*)*/*min (H(*a*),H(*b*)), where MI (*a, b*), *H* (*a*) and *H* (*b*) are local measures which are computed over a finite subblock in the floating and reference images. Note that the subblock size cannot be too small, we choose subblock size of 21 × 21 for local MI.

### 3.3. Quasi-Inverse Consistent Deformation Modeling

Although we have initialized correspondence detection in the context of local large deformation and outliers, it may not yet lead to 100% accurate matching of DoG control points, a few of the corresponding pairs are likely to be incorrect. We adopt an approximating [32, 43, 55] rather than interpolating RBF to remove the effects of those correspondence outliers on deformation modeling. Furthermore, a desirable property of inverse consistency for nonrigid registration should be considered in our work. That is, when the roles of reference image and floating images are swapped, the forward transformation *T*_1_ : **r***_i_* → **f***_i_*, is the inverse of the backward transformation *T*_2_ : **f***_i_* → **r***_i_*, *i.e.*, $T_{1}=T_{2}^{-1}$. Let {**r***_i_*} {**f***_i_*}, *i* = {1,…,*N*} denote a set of *N* reference and floating control points, the inverse consistent registration [16, 42] cost function is:

(15) $$D=\frac{1}{N}\sum_{i=1}^{N}\left\|{\text{r}}_{i}-T_{1}({\text{f}}_{i})\right\|+\frac{1}{N}\sum_{i=1}^{N}\left\|T_{2}^{-1}({\text{f}}_{i})-T_{1}({\text{f}}_{i})\right\|+J_{T}$$

where *T*_1_ is the RBF forward transformation, $T_{2}^{-1}$ is the inverse of the backward transformation. The first term measures the distance between the two control point sets and can be thought of as a registration accuracy constraint, and the second [16] measures inverse consistency at the point location, the third term represents the smoothness of the transformation and is formalized within the context of RBF transformation.

In this work we have used the Wendland's function as RBFs [43] to parameterize the transformation:

(16) $$R(d/\psi )=\{\begin{array}{ll} {\left(1-d/\psi \right)}^{4}(4d/\psi +1), & (1-d/\psi )>0\\ 0 & \text{else} \end{array}$$

where the *d* is a Euclidean distance and the *ψ* is a radius of support which defines the locality of the RBF deformation. The minimum *ψ* depends on the control point displacement Δ = max (Δ_1_, Δ_2_)(Δ_1_ and Δ_2_ are the displacement from the floating control point to the reference control point in *x* and *y* coordinate direction, respectively) with the constraint *ψ >* 2.98Δ.

Generally, an interpolation transformation function *T* : R^2^ → R^2^ for 2D images based on control points must fulfill the constraints *T* (**f***_i_*) = **r***_i_, i* = 1,…,*N*. Often, each coordinate of the transformation function is calculated separately, *i.e.*, the interpolation problem *T_l_* : R^2^ → R is solved for each coordinate *l* = 1, 2 with the corresponding constraints *T_l_* (**f***_i_*) = **r***_l,i_*. With *N* reference and floating control points in the globally registered images, the nonrigid registration could estimate the displacements for a floating points as follows [43]:

(17) $$T_{l}(\text{f})=\sum_{i=1}^{N}\beta_{i}R(\left\|\text{f}-{\text{f}}_{i}\right\|)$$

where the ‖**f** − **f***_i_*‖ is the Euclidean distance from **f** to **f***_i_*. *R*(*d*) = *R*(‖**d**‖) is the Wendland's radial basis function depending only on the Euclidean distance *d*. The RBFs *R*(‖**f** − **f***_i_*‖) are centered around the *N* control points. The RBFs coefficient *β_l_* = (*β_l,_*_1_,…*β_l,N_*)*^T^* can be calculated to maximize smoothness whilst interpolating through the control points by solving the following linear system:

(18) $$\beta_{l}={\left(\text{K}+\chi {\text{W}}^{-1}\right)}^{-1}{\text{r}}_{l}$$

where **r***_l_* = (**r***_l,_*_1_,…, **r***_l,N_*)*^T^* is an *N* vector of the *l*th coordinate of the reference points and **K** an *N* × *N* positive definite matrix (**K***_ij_* = *R*(‖**f***_i_* − **f***_j_*‖)). The matrix $\text{W}=\text{diag}\{1/\tau_{1}^{2},\ldots ,1/\tau_{N}^{2}\}$ and *τ_i_* represent point localization errors. *χ* is an approximating factor to control the tradeoff between the smoothness of deformation and the accuracy of control point matching, which is very important to maximize smoothness whilst preventing the folding or the tearing of the deformation field.

Nonrigid registration is started by the correspondence detection and the local deformation computation for the tumor resection area, subsequently modified by the surrounding clusters in the ascending order of *ζ_k_*. Nevertheless, the entire deformation field (especially the transition between each cluster's deformation field) would be non-smooth and even tearing, if the nonrigid registration is implemented once when the local correspondence detection and the local RBF-based deformation for each cluster are finished independently. Therefore, our method is processed within a deterministic annealing iteration framework (the maximum number of iterations is 5), both in terms of the inverse consistent correspondence detection as well as the approximating local transformation model.

Specifically, in the initial registration iteration, the search radius *δ_k_* of inverse consistent correspondence detection and the approximating factor *χ* in local RBF modeling are inversely proportional to the average JSM value *ζ_k_* of each cluster. With the process of registration, the contiguous area of the current cluster in floating image is locally changed by the previous iteration's and the neighboring cluster's local deformations, the template-matching based inverse consistent point correspondence should be adjusted around the current cluster before implementing the local RBF-based deformation. The correspondence search radius and the approximating factor values are gradually reduced, making the RBF-based local deformation field gradually moving from an approximation formulation to an interpolation formulation, thus effectively increasing the specificity of final correspondence detection and the smoothness of deformation fields.

## 4. Experimental Results

We preliminarily evaluated our algorithm on 5 pairs of pre- and intra-operative (or post-operative) MR T1 images that are corresponding slices of rigidly transformed 3D MR datasets. We first remove the skull near to the tumor resection area to prevent from introducing the deformation of rigid skull. We use 18 × 18 bins for 2D DoG keypoint joint histogram, which is exponentially smoothed with the exponential smoothing factor *ρ* being selected as 0.5 ∼ 0.6. A pseudo peak in the histogram is removed if a 30-pixel radius circular neighborhood has more than two neighboring peaks. We reject all the DoG keypoints that are more than 80 pixels away from any peaks in the histogram. In irregular setup of DoG control point grids and the correspondence detection, the maximum number of keypoints chosen as candidate control points in each cluster is equal to 30. With the process of registration in deterministic annealing framework, the search radius is iteratively reduced from 10 pixels to 4 pixels for the local MI-based correspondence detection. The optimal support radius *ψ* of RBF in this work is a fixed value of 60 while the approximating factor *χ* is iteratively reduced from 0.5 to 0.01. The registration is terminated when either the value of the cost function is below a predefined threshold (1 ∼ 1.5) or the maximum number of iterations is reached.

**Figure 3.**
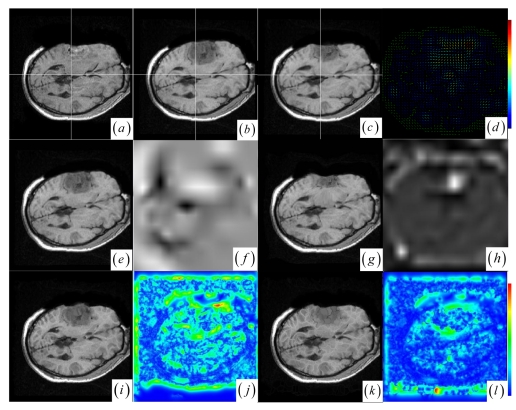
(a)–(b) Intra- and pre-operative MR images. (c) JKC. (d) Displacement vector field with the vector orientation and the variations of the vector color (the color scale encodes the norm of the displacement vector, in pixels). (e) BMI. (f) BMI deformation image. (g) BCR. (h) BCR deformation image. (i) DEM. (j) DEM deformation image (the color scale encodes the norm of the displacement vector, in pixels). (k) DIF. (l) DIF deformation image.

**Figure 4.**
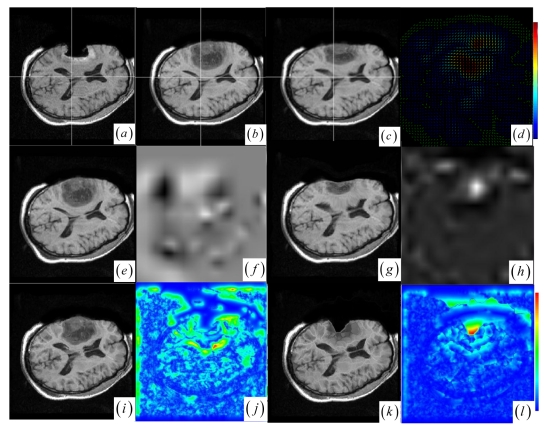
(a)–(b) Intra- and pre-operative MR images. (c) JKC. (d) Displacement vector field. (e) BMI. (f) BMI deformation image. (g) BCR. (h) BCR deformation image. (i) DEM. (j) DEM deformation image. (k) DIF. (l) DIF deformation image.

**Figure 5.**
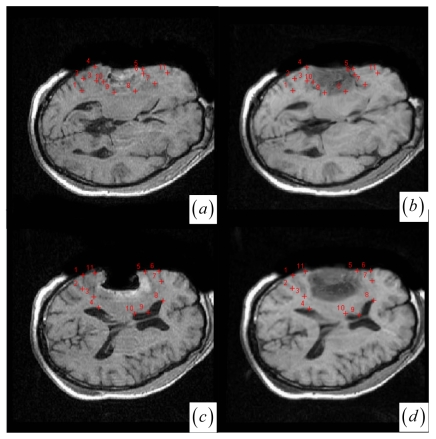
(a)–(b) Corresponding landmarks for intra- and pre-operative MR images at Figure 3(a)–(b). (c)–(d) Corresponding landmarks for intra- and pre-operative MR images at Figure 4(a)–(b).

In contrast to four state-of-the-art intensity-based registration methods, including B-Spline with correlation ratio [56] (BCR), B-Spline with normalized MI (BMI) [34, 57], Demons (DEM) [58] and diffeomorphic Demons (DIF) [59], which are failed or unsatisfactory in tumor resection with outliers and local large deformation, our proposed method based on JSM & keypoint clustering (JKC) successfully model the tumor resection-induced brain shift (see Figures 3–4 and Table 1 cases 1–3). Table 1 summarizes the registration quality in terms of correlation ratio (CR) and normalized MI (NMI) [4, 60]. Figure 3a–d and Figure 4a–d show that the regions around the tumor areas have smoothly deformed to shift towards the tumor resection areas in the JKC registration results. The smoothly constructed deformation field is displayed by means of displacement vector field with variations of the vector color (from blue to red according to the displacement amplitude in pixels). The quality of these registration results also can be validated by the respective small CRs listed in the Table 1 cases 1–3. We also define the average error distance between the manually defined reference landmarks and the floating landmarks in the registered images (Figure 5 shows that most landmarks are around the tumor resection areas and the neighboring normal tissues), our method's average accuracy for the three cases of local large deformation (cases 1–3) achieves an average error distance of less than 1.2 pixel while the average accuracy for the two cases of small deformation achieves an average error distance of less than 1 pixel.

The BCR and BMI registration are implemented at two pass with the different transformation options (B-Spline degree for all axes: 1, 2; B-Spline control points for all axes: 8, 16; gradient descent minimize step size: 1.0, 0.5; gradient descent minimize maximum search steps: 10, 10) and the different iteration options (the convergence limit of minimum change rate for one iteration: 0.1, 0.01; maximum number of iterations: 10, 10) [57]. The dark areas on the deformation image (Figure 3f,h and Figure 4f,h) are related to the areas on the two images which did not perform a deformation during the registration or the deformation was relatively small. The light areas are related to the areas on the two images which perform a bigger deformation. In contrast to the BMI (Figure 3e,f and Figure 4e,f) that fails to compensate the local large deformation around the tumor areas, the BCR (Figure 3g and Figure 4g) can guide the anatomical structure deformation around the tumor resection regions (This also can be validated from the cases 1–3 at Table 1, at which the BCR have smaller CRs than the BMI, DEM and DIF). Nevertheless, the BCR has blurred and excessively shrunk the pre-operative anatomical structures around the tumor areas. From this experiment, we also confirm that the CR is more appropriate to be a similarity measure for the nonrigid monomodal registration of small structures than the NMI.

The DEM and DIF registration are conducted with a maximum step length of 2 pixels, 1.0 standard deviations of the Gaussian smoothing, a maximum number of 200 iterations and 0.001 intensity difference threshold. Treating each image as a set of iso-intensity contours and assuming the same anatomical point having the same intensity level in both images, the DEM and DIF easily distort the data to some extent, which may introduce strange artifacts similar to pieces of small mosaic patterns in the deformed pre-operative images (Figure 3i,k and Figure 4i,k). Additionally, the ”demons algorithm” with its large number of degrees-of-freedom allows to run into problems with the physical fidelity of the deformation field (Figure 4k and the DIF at case 2 in Table 1).

**Figure 6.**
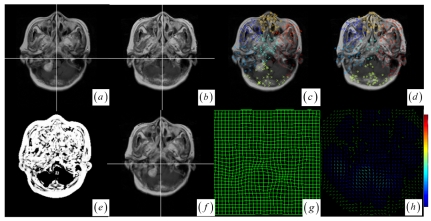
(a)–(b) The pre- and post-operative brain tumor images. (c)–(d) Significant keypoints selected from the different clusters. (e) JSM between the two images. (f) Fused image using check pattern after registration. (g) Warped mesh after registration. (h) Displacement vector field.

**Figure 7.**
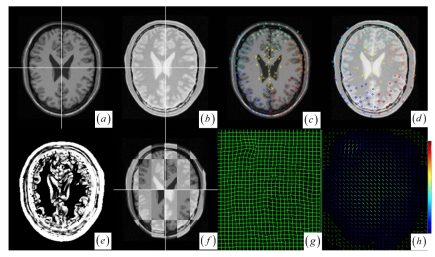
(a)–(b) The MR-T1 and MR proton density weighted images. (c)–(d) Significant keypoints selected from the different clusters. (e) JSM between the two images. (f) Fused image using check pattern after registration. (g) Warped mesh after registration. (h) Displacement vector field.

In the two cases of local small structure distortion, all five methods get good results (see Figure 6 and Table 1 cases 4–5). To facilitate the visual assessment of registration accuracy, we also use a mosaic (or checkerboard) pattern to fuse the two images. Figure 6a–b display the multi-temporal brain changes with a small tumor presented in the preoperative MRI image but not in the post-operative MRI image. From clustering analysis, the brain images have seven clusters (Figure 6c,d) with no cluster's average JSM (Figure 6e) value less than the average JSM threshold, so that there is no need for any cluster to reject outliers before registration procedure. Although there exist a few outlier keypoints in the small tumor, the clustering-based correspondence detection and the approximating RBF modeling could suppress the effect of outlier keypoints on registration result (Figure 6f). We also applied the method to multimodal brain image registration. Figure 7a–b show the T1 MRI (reference image) and proton density MRI (floating image) distorted by known deformations, which have six clusters with average JSM (Figure 7c–e) values {0.400, 0.556, 0.631, 0.652, 0.687, 0.741}. There is no need for any cluster to reject outliers in locally distorted structures.

## 5. Conclusions

We have presented a new hybrid nonrigid registration of brain MRI images to model tumor resection-induced brain shift. While SIFT keypoint detector was designed under the assumption of linear changes in intensity, the DoG keypoint detected by the SIFT detector can be effective in robustly matching intra- and pre-operative MR image pairs taken under substantially different illumination condition due to the spatially-varying intensity inhomogeneity and large intra-operative noise. Nevertheless, the keypoint detection (and the keypoint description) could not sufficient [28] for the unambiguous correspondence detection in the nonrigid image registration with the complex local large deformation and outliers, and should be incorporated with the structural mismatch detection mechanism and other useful correspondence constraints. By testing on a few typical clinical images and comparing four state-of-the-art nonrigid registration methods, this work introduces the JSM and keypoint clustering towards the robust keypoint correspondence detection for nonrigid registration of brain tumor resection MR images.

The main drawback of our approach is that a number of parameters should be set. By applying more experiments on the brain tumor resection images, the future study must be carried out determining the optimal parameters and comparing the JSM reconstruction methodologies with discussion their robustness. An interesting future development is to design a robust deformation invariant keypoint detector [29] which should meet both needs for reconstructing JSM to detect the structural mismatches and finding good landmark with high repeatability for nonrigid registration.

## Figures and Tables

**Table 1.** CR and NMI values for different registration methods (The smaller values usually mean the better registration results).

cases	CR					NMI				

	JKC	BCR	BMI	DEM	DIF	JKC	BCR	BMI	DEM	DIF
1	0.0613	0.0553	0.1068	1.0000	0.9998	0.8619	0.8595	0.8713	0.9845	0.9846
2	0.0889	0.0743	0.2167	0.0974	0.0349	0.8607	0.8590	0.8775	0.8495	0.8195
3	0.0695	0.0704	0.2673	0.9354	0.8917	0.8421	0.8611	0.8697	0.9783	0.9624
4	0.0637	0.0974	0.4181	0.0590	0.0649	0.8745	0.8239	0.8580	0.7880	0.7925
5	0.0749	0.0858	0.3177	0.0582	0.0638	0.8657	0.8471	0.8693	0.7919	0.8142
